# TRIM8: a double-edged sword in glioblastoma with the power to heal or hurt

**DOI:** 10.1186/s11658-023-00418-z

**Published:** 2023-01-23

**Authors:** Hamed Hosseinalizadeh, Omid Mohamadzadeh, Mohammad Saeed Kahrizi, Zahra Razaghi Bahabadi, Daniel J. Klionsky, Hamed Mirzei

**Affiliations:** 1 Department of Medical Biotechnology, Faculty of Paramedicine, Guilan University of Medical Sciences, Rasht, Iran; 2 Department of Neurosurgery, Tehran University of Medical Science, Tehran, Iran; 3 Department of Surgery, Alborz University of Medical Sciences, Karaj, Alborz Iran; 4 School of Medicine, Kashan University of Medical Sciences, Kashan, Iran; 5 Student Research Committee, Kashan University of Medical Sciences, Kashan, Iran; 6 Life Sciences Institute and Department of Molecular, Cellular and Developmental Biology, University of Michigan, Ann Arbor, MI USA; 7 Research Center for Biochemistry and Nutrition in Metabolic Diseases, Institute for Basic Sciences, Kashan University of Medical Sciences, Kashan, Iran

**Keywords:** Autophagy, Glioblastoma, JAK-STAT, NF-κB, p53, Stem-cell, TRIM8

## Abstract

Glioblastoma multiforme (GBM) is an aggressive primary brain tumor and one of the most lethal central nervous system tumors in adults. Despite significant breakthroughs in standard treatment, only about 5% of patients survive 5 years or longer. Therefore, much effort has been put into the search for identifying new glioma-associated genes. Tripartite motif-containing (TRIM) family proteins are essential regulators of carcinogenesis. TRIM8, a member of the TRIM superfamily, is abnormally expressed in high-grade gliomas and is associated with poor clinical prognosis in patients with glioma. Recent research has shown that TRIM8 is a molecule of duality (MoD) that can function as both an oncogene and a tumor suppressor gene, making it a “double-edged sword” in glioblastoma development. This characteristic is due to its role in selectively regulating three major cellular signaling pathways: the TP53/p53-mediated tumor suppression pathway, NFKB/NF-κB, and the JAK-STAT pathway essential for stem cell property support in glioma stem cells. In this review, TRIM8 is analyzed in detail in the context of GBM and its involvement in essential signaling and stem cell-related pathways. We also discuss the basic biological activities of TRIM8 in macroautophagy/autophagy, regulation of bipolar spindle formation and chromosomal stability, and regulation of chemoresistance, and as a trigger of inflammation.

**Graphical Abstract:**

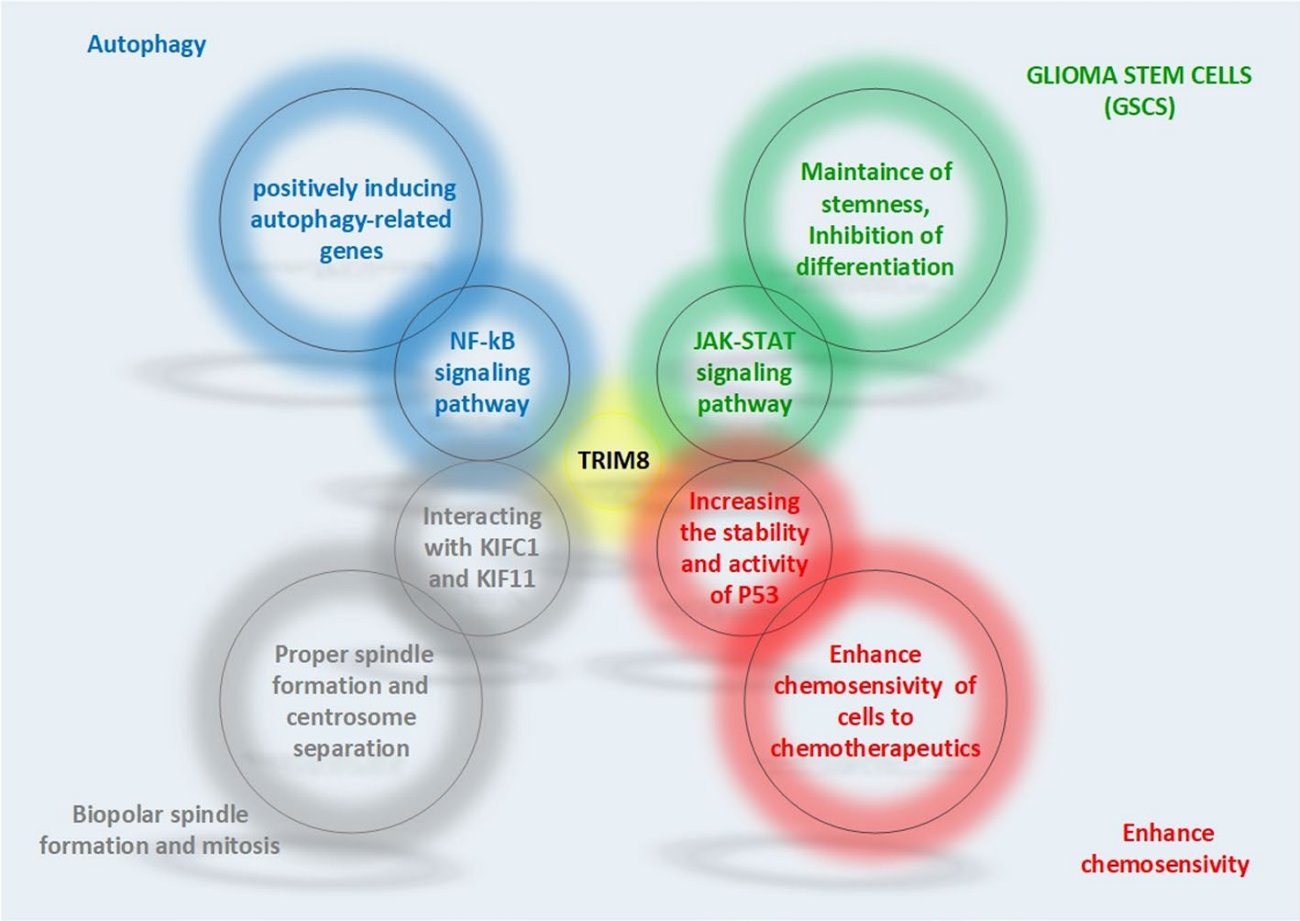

## Introduction

Glioblastoma (GBM) is a highly lethal brain tumor that can arise from astrocytes, a type of brain cell [1, 2]. Despite recent advances in diagnosis and treatment, the prognosis for patients with late-stage GBM is abysmal. The median survival is 14–17 months when treated with conventional multimodal therapy, which primarily includes surgery followed by chemotherapy, radiotherapy, and sometimes steroid therapy [3, 4]. The failure of these treatments is a direct consequence of glioma stem cells that are highly resistant to standard chemotherapy and radiation, the large intratumoral and intertumoral diversity that significantly reduces the efficacy of targeted agents, and the dysregulated cellular metabolism that has evolved to take advantage of the nutrient-rich environment of the central nervous system [4]. Therefore, it is crucial to understand better which signaling pathways or molecular alterations promote GBM tumor progression to develop new therapeutic strategies for early diagnosis and targeted therapy to improve the prognosis of patients with GBM [5, 6]. Members of the tripartite motif protein family (TRIM) are E3 ubiquitin (Ub) ligases characterized by the presence of three conserved domains known as RBCC (RING domain, B-box motif, and coiled-coil domain) [7]. TRIM proteins can regulate various biological processes, including viral restriction, cell cycle regulation, DNA repair, apoptosis, stress response, protein quality control, and autophagy [8]. It is, therefore, not surprising that their altered expression correlates with many adverse conditions, including congenital abnormalities and a higher risk of tumorigenesis [7]. Within the large family of TRIM proteins, TRIM8 is a well-characterized member of the ubiquitin-related protein family and is found to operate as an oncogene or tumor suppressor, serving as a “double-edged sword” [9]. In addition to the RBCC domains, TRIM8 protein contains a nuclear localization signal (NLS) that is required for nuclear localization. The TRIM8 coiled-coil domain enables the formation of nuclear bodies (NBs), which are important interchromatin structures, implying that TRIM8 regulates the function of important cellular proteins via protein–protein interactions [10]. The *TRIM8* gene is located on chromosome 10q24.32, an area known to have extensive deletion or loss of heterozygosity in 88% of GBMs. However, this deletion does not result in a reduction in TRIM8 protein, leading to the alternative name GERP (glioblastoma-expressed RING finger protein) for the gene product [11]. Few studies have been performed to thoroughly understand the activities and underlying mechanisms of TRIM8 in GBM [12]. TRIM8 can respond to various stimuli, including genotoxic stress and viral or bacterial attack. Moreover, TRIM8 is critical in many biological processes, including cell survival, innate immune response, carcinogenesis, autophagy, apoptosis, differentiation, and inflammation. TRIM8 has either a tumor suppressive or an oncogenic function regulating the proliferation of GBM cells. The importance of TRIM8 in modulating the p53 tumor suppressor pathway indicates that it plays a tumor-suppressive role in GBM. In contrast, a number of oncogenic mechanisms have been proposed for TRIM8, as it is involved in the positive regulation of NF-κB (nuclear factor kappa-light-chain-enhancer of activated B cells) and JAK-STAT signaling pathways, promoting tumor development and progression [13]. Brat et al. demonstrated that upregulation of TRIM8 in adult tissues and a variety of tumors, particularly GBM, correlates with higher-grade cancer, massive tumor size, and increased stem cell formation and self-renewal ability of cancer stem cells (CSCs) [14]. Regarding TRIM8 tumor suppressor activity, Micale et al. showed that restoring TRIM8 expression in patient glioma cell lines inhibits tumor development and significantly reduces clonogenic potential [15].

In the present review, we sought to elucidate both the tumor suppressive and oncogenic functions of TRIM8 and the underlying molecular networks in GBM. Our findings contribute to a better understanding of TRIM8 and provide clues for developing a new approach to the treatment of GBM cancer.

## TRIM8 acts as a novel marker for malignant glioma stem cells

GBM is the most common and lethal type of primary brain tumor, even after treatment with standard therapies. Over the past decade, glioblastoma stem cells (GSCs) have been extracted from GBM and characterized. These cells have likely played a critical role in tumorigenesis and therapy resistance due to their unique properties, such as self-renewal and pluripotency, suggesting that GSCs are a new effective target for treatment [16]. Therefore, searching for regulators that effectively enhance the stem-like property of GSCs may provide clues for innovative treatments. Zhang et al. reported that the expression of TRIM8 is consistently correlated with stem cell markers or other transcription factors such as PROM1/CD133, NES (nestin), SOX2, and MYC/c-MYC, and partially correlated with OLOG2 and NANOG, and therefore could promote stem cell property in GBM [14]. They observed that overexpression of TRIM8 results in increased expression of these stem cell markers and transcription factors involved in the expression of two distinct groups of genes: those engaged in tumor dedifferentiation status and stemness acquisition [9, 14]. They also found significant MKI67/Ki-67 protein expression in GSCs overexpressing TRIM8 [14]. MKI67 is a protein commonly used as a cell proliferation marker, and its increased expression in human cancer tissues is closely associated with worse histological grade [17]. They concluded that overexpression of TRIM8 not only correlates with the expression of stem cell markers and transcription factors in GSCs but also increases stem cell activity. Knockdown of TRIM8 inhibits self-renewal of GSCs, and the expression of stem cell markers and transcription factors such as NES, SOX2, and, to a lesser extent, PROM1/CD133 and MYC are significantly impaired. The expression of MKI67 is also reduced, suggesting lower cell proliferation after TRIM8 downregulation [14].

Further investigation of the mechanisms behind the effect of TRIM8 on stem cell maintenance and the ability of GSCs to self-renew revealed that TRIM8 acts mainly by stimulating the JAK-STAT signaling pathway [14, 18]. Protein inhibitor of activated STAT (PIAS) plays a crucial role in regulating the balance and steady state of signal transducer and activator of transcription (STAT) by decreasing the activity and translocation of this protein [19]. PIAS3 specifically interacts with phosphorylated STAT3 via the latter’s DNA-binding domain, thereby inhibiting its physical binding to target genes [20, 21]. Activated STATs are critical regulators of GSCs and are involved in various physiological processes, including immortalization and inhibition of differentiation. TRIM8 interacts with PIAS3 and inhibits its activation, either by degrading PIAS3 through the ubiquitin–proteasome machinery or by significantly reducing its nuclear translocation, resulting in enhanced STAT3-mediated support of stem cell properties in GSCs.

**Fig. 1:**
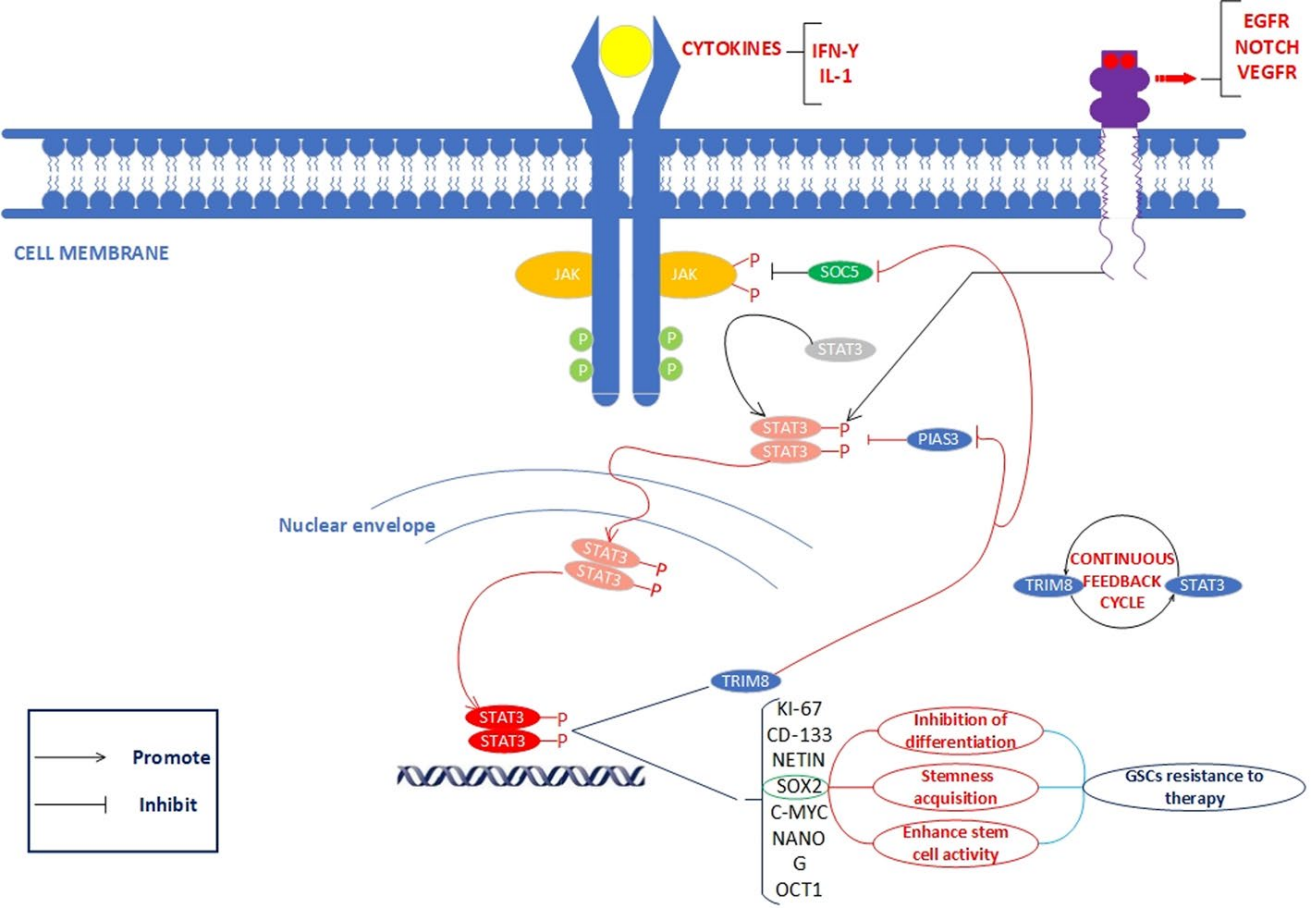
Schematic representation of the TRIM8-PIAS3-STAT3 pathway, which leads to therapeutic resistance in GSCs. TRIM8 induces ubiquitin-mediated proteasomal degradation of PIAS3 and SOCS1, contributing to activation of STAT3 and subsequent promotion of expression of GSC-related markers and transcription factors, including MYC, SOX2, PROM1/CD133, POU2F1/OCT1, NANOG, MKI67, and NES. These markers and transcription factors play a functional role in stem cell acquisition, inhibition of differentiation, and enhancement of stem cell activity in this tumor type. In addition, STAT3 increases TRIM8 expression, leading to a positive TRIM8-STAT3 feedback loop in GBM. This offers new insight into how targeting TRIM8 or STAT3 could effectively affect GBM self-renewal and tumor growth

STAT3 is an essential regulator of normal stem cells and cancer stem cells; it mainly transmits signals from cytokine-stimulated receptors in the plasma membrane through interactions with importins into the nucleus, where they regulate gene expression directly or indirectly via other transcription factors involved in maintaining undifferentiated phenotype in stem cells and cancer stem cells [22]. STAT3 exerts its effect on GSCs by binding to and inducing the expression of promoters of genes encoding transcription factors essential for maintaining self-renewal or pluripotency, such as SOX2, POU2F1/OCT1, NES, PROM1/CD133, and MYC [23, 24]. Studies have shown that the knockdown of TRIM8 inhibits stem cell formation and self-renewal capacity of GBM and leads to glial differentiation. Moreover, STAT3 promotes the expression of TRIM8, resulting in a positive continuous feedback cycle between TRIM8 and STAT3 [18]. The discovery of the positive TRIM8-STAT3 feedback cycle in GSCs sheds new light on the possibility of disrupting the positive feedback loop by targeting either TRIM8 or STAT3 and opens new opportunities for developing treatments that affect pluripotency in GSC and other malignancies with TRIM8 overexpression (Fig. 1) [25]. Further research is needed to understand the pathways that lead to increased *TRIM8* transcription in response to STAT3 activation. The *TRIM8* promoter contains two potentially conserved STAT3 binding sites and several MYC and POU2F1/OCT1 transcription binding sites [14]. Therefore, STAT3 could either directly or indirectly activate *TRIM8* transcription via MYC and POU2F1/OCT1 [26, 27].

In addition to positively regulating the JAK-STAT signaling pathway in GSCs, TRIM8 also positively regulates the NF-κB signaling pathway. The NF-κB pathway activates the expression of GSC-associated genes such as CD44/HCAM, SOX2, and NANOG. TRIM8, through its role as a crucial activator of NFKB, enhances signaling pathways initiated by proinflammatory cytokines such as TNF/TNFα (tumor necrosis factor) and IL1B/IL-1β (interleukin 1 beta) [28, 29]. In particular, TNF-induced NFKB activation is a critical regulator of cell survival and apoptosis, which has implications for various physiological and pathological conditions, including cancer [28]. Li et al. demonstrated that TRIM8 mediates K63-linked polyubiquitination of MAP3K7/TAK1 (mitogen-activated protein kinase kinase kinase 7) at the K158 residue, which is associated with MAP3K7/TAK1 activation in TGFB/TGFβ signaling [30]. Subsequently, activated MAP3K7/TAK1 led to phosphorylation and degradation of NFKBIA/IκBα, an essential NFKB inhibitor protein [9, 30]. Once NFKBIA is degraded, the NFKB transcription factor translocates to the nucleus. It promotes the expression of key stem cell transcription factors that ultimately mediate fundamental elements of GSC biology, including self-renewal, proliferation, and metastasis, either alone or in collaboration with other signaling pathways.

Tomar et al. identified another possible mechanism by which TRIM8 triggers NF-KB activation. PIAS3 inhibits NFKB-dependent transactivation by binding to RELA/p65 and affecting the transcriptional activity of RLA/p65 in the nucleus. The SUMOylation of endogenous RelA by PIAS3 mediates negative regulation of the NF-κB transcription factor [28]. TRIM8 interacts with PIAS3 and mediates its transport from the nucleus to the cytoplasm and its degradation [31]. Nucleocytoplasmic translocation of TRIM8 is required for positive control of NFKB activation [28]. Tomar et al. have observed the function of TRIM8-induced NFKB regulation and its nuclear localization for the migratory and clonogenic abilities of HEK293 cells [28]. This finding of TRIM8-mediated enhancement of cell motility and clonogenic capacity needs further investigation, as it may provide important information about the role of TRIM8 and establish links between inflammatory responses and cancer [18, 32, 33].

The pathways that stimulate TRIM8 or induce its enhanced expression in GSCs are unclear. Toniato et al. showed that TRIM8 interacts with SOCS1 (suppressor of cytokine signaling 1) both in vitro and in vivo. This association requires the SH2 domain and the SOCS box of SOCS1 [34]. This interaction decreases the stability and abundance of the SOCS1 protein, resulting in decreased suppression of IFN-induced JAK-STAT activation. As described previously, specific cytokines, such as IFNG/IFN-γ and IL1, can increase *TRIM8* mRNA expression via a positive continuous feedback cycle between STAT3 and TRIM8 [35, 36]. The mechanism by which SOCS1 decreases JAK-STAT signaling is partially known. The SOCS protein family plays a critical negative regulatory role in cytokine-mediated JAK kinase signaling [37]. SOCS proteins can interfere with cytokine signaling through two distinct pathways. They serve as ubiquitin ligases for ubiquitination-dependent regulation of signaling components or directly inhibit JAK tyrosine kinase receptors via their kinase inhibitory domains (KIRs) [37, 38]. Interleukin 6 (IL6) is another potent trigger of TRIM8 in GSCs. Abnormal IL6 production and signaling significantly increase STAT3 activity in GSCs, and recent research shows that this is closely linked to their ability to self-renew via binding to the IL6R/IL-Rα receptor. Thus, IL6 increases TRIM8 expression in GSCs via STAT3 activation [39]. Interestingly, increased IL6 levels lead to a dose-dependent increase in TRIM8 protein expression. Other cognate receptors and associated signaling cascades that promote STAT3 activation in GSCs, such as EGFR, NOTCH, and KDR/VEGFR, may also enhance TRIM8 in GSCs through positive interactions between TRIM8 and STAT3 [40, 41].

## TRIM8, a double-edged sword in glioblastoma

In contrast to previous data demonstrating the oncogenic role of TRIM8 in GSCs, studies now also indicate the tumor-suppressive function of TRIM8 in GBM. Micale et al. showed that TRIM8 expression is significantly decreased in patients with glioma at high risk of mortality and higher risk of disease progression. The expression of TRIM8 in grade IV gliomas is considerably lower than in grade III gliomas, indicating a negative correlation with higher-grade GBM [15, 42]. The authors observed that overexpression of TRIM8 decreases cell proliferation by approximately 25% and results in a significant reduction in clonogenic potential as an indirect indicator of tumorigenic potential, suggesting that TRIM8 has proliferation inhibitory properties in patients with glioma [43]. A possible target of *MIR17* is TRIM8, which regulates TRIM8 expression at the transcriptional and posttranscriptional levels. Suppression of *MIR17* significantly decreases cell viability and enhances apoptotic activity in glioma cell lines, and overexpression of this miRNA has been associated with accelerated tumor growth and poor overall survival in gliomas [44]. Thus, these findings suggest a feedback loop between *MIR17* and TRIM8 [15, 43].

Okumura et al. showed that TRIM8 binds to HSP90 in embryonic stem cells and specifically inhibits transcription of NANOG, a master regulator of pluripotency, by preventing excessive signal transduction via STAT3 [45]. HSP90, a molecular chaperone, is one of the endogenous binding partners of TRIM8 and facilitates the translocation of activated STAT3 into the nucleus. In stem cells, TRIM8 suppresses the translocation of the HSP90-STAT3 complex into the nucleus, modulating *Nanog* transcription but not that of other transcription factors such as POU5F1/OCT3/OCT4 and SOX2 via STAT3 [46]. Suppression of TRIM8 increases transcription of *Nanog* in mouse embryonic stem (ES), suggesting that TRIM8 plays an essential role in controlling STAT3-mediated signaling in ES cells. In contrast, the expression of TRIM8 results in the spontaneous differentiation of stem cells [45, 46]. Therefore, TRIM8 is thought to be a dual positive and negative regulator of stem cell properties, and its expression must be tightly regulated at an appropriate level to maintain stem cell pluripotency. It is still unclear how TRIM8 can maintain GSC self-renewal capacity and alter its mechanism to reduce glioma cell proliferation and clonogenic potential.

Recently, TRIM8 was found to be involved in several cellular signaling pathways critical in cancer suppression. The tumor suppressor and transcription factor TP53/p53 is one of the most critical factors in controlling cell proliferation and is deregulated in nearly 84% of patients with GBM. The tumor suppressor TP53 modulates the expression of genes involved in cell cycle arrest, DNA damage response, and programmed cell death (apoptosis) [47]. Under various stress conditions, such as UV radiation or genotoxic stress, TP53 directly targets the *TRIM8* gene and induces its expression. In a positive feedback loop, TRIM8 interacts with TP53 and impairs its interaction with MDM2, a negative regulator of TP53, thereby increasing TP53 stability [48, 49]. TRIM8-stabilized TP53 mediates G1 cell cycle arrest through increased expression of CDKN1A/p21 and GADD45 [48]. At the same time, TRIM8 induces polyubiquitination and degradation of MDM2, which further promotes TP53-dependent cell growth arrest [49]. This suggests that TRIM8 not only plays a role in enhancing the efficacy of chemotherapeutic agents by reactivating the TP53 pathway but may also be an alternative pathway to increase TP53 activity in malignant cancers; thus, an increase in TRIM8 expression could be used as an enhancer of chemotherapy efficacy in a TP53 wild-type background [50].

Other studies by Mastropasqua et al. have expanded the understanding of the molecular mechanisms underlying the downregulation of TRIM8 in oncogenesis and chemoresistance [51]. The authors found that TRIM8 is negatively associated with *MIR17-5p* and *MIR106B*-*5p*, both of which are overexpressed in many different chemo/radioresistant cancers, resulting in a lack of TP53 protein activation by disrupting the positive feedback loop between TRIM8 and TP53 [51]. The oncoprotein MYCN/N-MYC, typically overexpressed in GBM, stimulates MIR17-5p and MIR106B-5p transcription, highlighting its role as an oncogene. Along these lines, activation of TRIM8 in TRIM8-deficient cells improves the efficiency of chemotherapy in resistant cancer cell lines. This occurs not only by reactivating the tumor suppressor function of TP53 but also by enhancing the transcription of *MIR34A*, which suppresses the activity of MYCN [52, 53]. As a result, these miRNAs no longer silence TRIM8. However, in other cases, simultaneous activation of TRIM8 and TP53 may lead to adverse effects, such as in response to hypoxic stress caused by ischemia after stroke or myocardial infarction [54]. Recent findings in clear cell renal cell carcinoma (ccRCC) have shown that a higher percentage of wild-type TP53 is present in most aggressive drug-resistant cell lines, highlighting the significant association between TRIM8 deficiency, TP53 inactivation, and chemoresistance. Restoration of TRIM8 expression in ccRCC cell lines decreases cell growth rate in a TP53-dependent manner.

Interestingly, restoration of TRIM8 expression makes the cells more susceptible to therapy with axitinib and sorafenib, two specific drugs now used to treat a variety of malignancies, including ccRCC [48, 50]. However, another study found that suppression of TRIM8 in Ewing sarcoma cells increases DNA damage and makes the cells susceptible to DNA damage inhibitors such as olaparib. More in-depth research on TRIM8-mediated regulation of TP53 activity or its anti-cancer capacity will significantly enhance our understanding of the complex framework based on TP53 dynamics and provide better insight into the ability of TRIM8 to restore the native conformation of TP53 mutants and reactivate its tumor-suppressor function.

In cancer cells, chromosomal abnormalities often lead to increased transcription factors (TFs) activity and form a class of driving oncoproteins that are difficult to target effectively. Recent research has shown that TRIM8 plays a vital role in the degradation of certain oncoproteins [55, 56]. Stegmaier et al. found that TRIM8 degrades the EWS/FLI oncoprotein, a driving fusion TF in Ewing sarcoma, and is associated with improved overall survival. Ewing sarcoma is defined by a genome translocation combining the EWSR1 transactivation domain with the FLI1 DNA-binding domain. EWS/FLI is a TF that recruits chromatin remodeling complexes such as the BAF complex to gain access to packed chromatin [57]. The results of Stegmaier et al. have shown that EWS/FLI can be indirectly targeted by TRIM8, opening a new therapeutic window for treating Ewing sarcoma by targeting TRIM8 [55].

TRIM8 is involved in a number of critical cellular processes, including carcinogenesis, autophagy, innate immunity, apoptosis, differentiation, and inflammatory responses, and is closely related to DNA repair, metastasis, tumor suppressive regulation, and carcinogenic regulation. In the following section, we review some essential cellular processes in which TRIM8 has tumor suppressive or oncogenic functions. These include autophagy, regulation of bipolar spindle formation and chromosomal stability, regulation of chemoresistance, and induction of inflammation.

### Autophagy

Autophagy supports cellular fitness by directing poorly functioning proteins, damaged DNA, aggregates, and damaged organelles to lysosomes for degradation, and it is critical for providing energy and macromolecular precursors for cancer cell progression [58–61]. TRIM8 is particularly important in cancer, where autophagy promotes and inhibits tumor growth. TRIM8 is emerging as a critical regulator of cell survival under various genotoxic stress conditions by promoting autophagy flux and regulating lysosomal biogenesis [9, 62]. This function can improve cancer cell survival under genotoxic stress conditions by allowing cells to repair DNA damage through autophagy, reducing cytotoxicity, and protecting cells from the cell death response after DNA damage so that cell repair and proliferation can continue [13]. Autophagy triggered by genotoxic stress plays an essential role in cell survival and death. Roya et al. have shown that TRIM8 is stable and exhibits a high turnover under genotoxic stress conditions [62]. TRIMs form homo- and hetero-oligomers with other TRIMs and become either indirectly ubiquitinated via their binding partner or directly ubiquitinated due to their innate ubiquitin ligase activity, modulating their turnover under various pathophysiological conditions [62, 63].

**Fig. 2:**
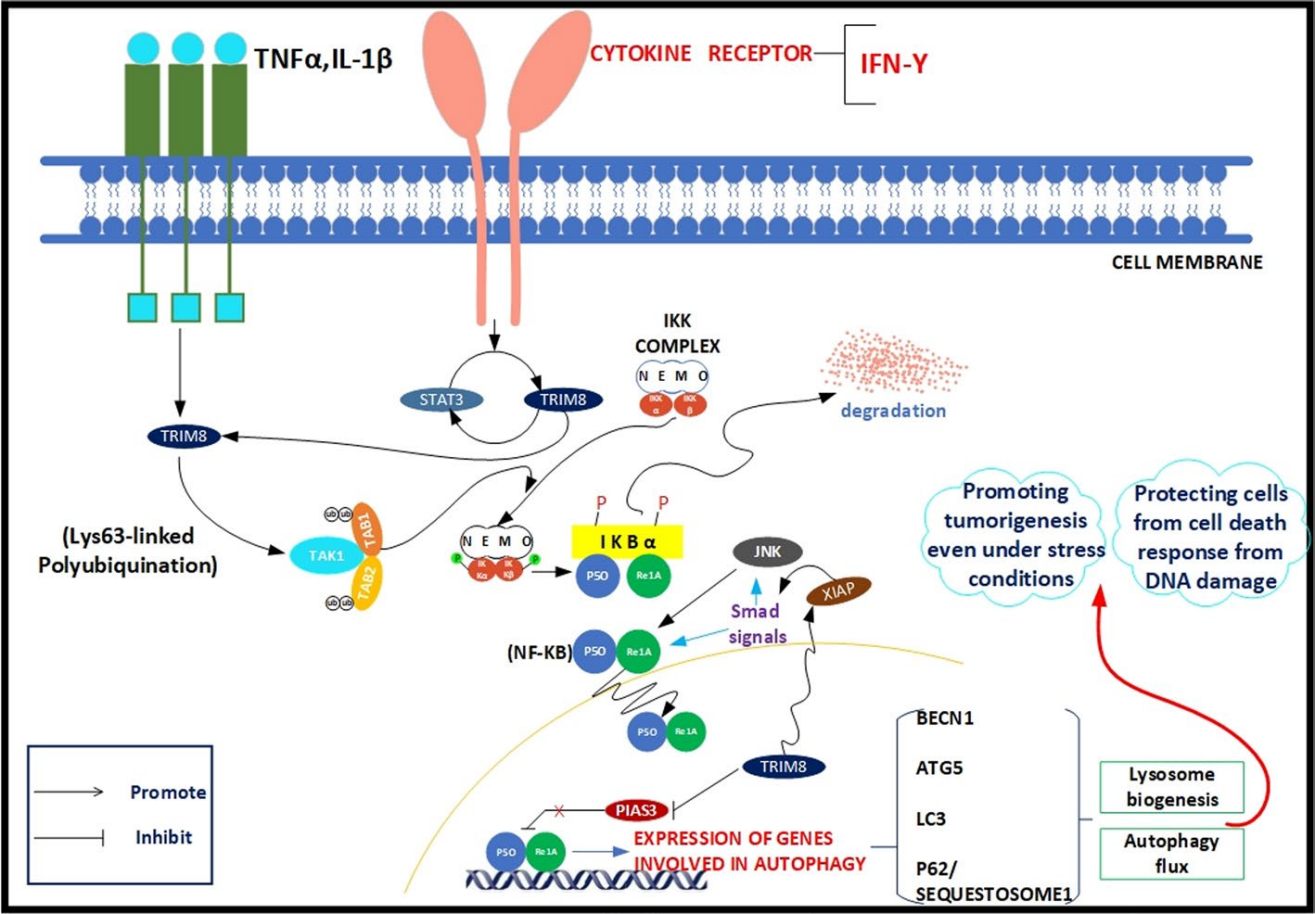
Schematic representation of the oncogenic activity of TRIM8 as a positive regulator of TNF- and IL1B-triggered NFKB and transcriptional induction of genes involved in autophagosome formation. The TNF and IL1 family of cytokines are the best-characterized triggers of autophagy through activation of NFKB. TRIM8 is important for TNF- and IL1B-induced NFKB activation by interacting with MAP3K7/TAK1, which is required for NFKB activation, and mediating K63-linked polyubiquitination. The MAP3K7/TAK1-TABs complex phosphorylates IKBKB/IKKβ and CHUK/IKKa, which further activate the NFKB transcription factor by phosphorylating and degrading NFKBIA/IκBα (NFKB inhibitor alpha). Once degraded, the NFKB dimer (RELA/p65-NFKB1/p50 subunits) translocates to the nucleus, where it binds to the DNA consensus sequence of genes involved in autophagy induction, such as *BECN1*, *ATG5*, *MAP1LC3/LC3*, and *SQSTM1/p62*. These genes are involved in lysosome biogenesis and autophagy flux, which in cancer cells promote tumorigenesis even under stress conditions and protect cells from cell death due to DNA damage. TRIM8 also counteracts the negative effect of PIAS3 on NFKB through polyubiquitination and degradation of PIAS3. Upon genotoxic stress, TRIM8 stabilizes XIAP, leading to activation of NFKB via the activating SMAD and MAPK/JNK signaling pathways. As a result, apoptosis in cancer cells is suppressed and cell growth is positively controlled. Cytokines such as IFNG and IL1 increase TRIM8 expression through activation of the JAK- STAT pathway and due to the existence of a continuous positive feedback loop between STAT3 and TRIM8

The ubiquitin ligase activity of TRIM8 via its RING domain as a posttranslational modification is required to regulate autophagy pathways positively. For instance, polyubiquitin chains linked via the Lys63 residue of ubiquitin are involved in the signaling cascades associated with autophagy and recruit several ubiquitin-binding proteins such as IKBKG/NEMO, the regulatory subunit of the IκB kinase (IKK) complex. These ubiquitin-binding proteins are necessary for TNF- and IL1B-mediated NFKB activation [64, 65]. TRIM8 indirectly modulates transcription of autophagy-regulating genes via activation of NFKB. NFKB transcription factors have been shown to be crucial triggers of autophagy and can trigger this process by inducing the expression of genes or proteins involved in the machinery that generates phagophores, such as BECN1, ATG5, and MAP1LC3/LC3 [66, 67]. Interestingly, TRIM8 may indirectly regulate the level of SQSTM1/p62 (sequestosome 1), a pleiotropic protein that functions as a selective autophagy receptor and promotes mitophagy, thereby promoting tumorigenesis [68, 69]. These results highlight the intricate interaction between the TRIM8-mediated regulation of SQSTM1/p62 and its potential function beyond autophagy and cancer. Etoposide, a genotoxic agent, causes apoptosis through the involvement of the effector caspase 3 (CASP3) [70]. Studies have shown that autophagy regulated by TRIM8 can induce the degradation of activated CASP3, one of the significant cysteine proteases of the apoptotic cascade, to prevent cell death caused by genotoxic stress [62]. In addition, TRIM8 regulates cell death and autophagy by stabilizing XIAP/IAP3 (X-linked inhibitor of apoptosis) [71]. XIAP can interrupt both the “extrinsic” and “intrinsic” death pathways by directly inhibiting the proteolytic activity of CASP9 and the effectors CASP3 and CASP7 via its BIR domains. XIAP forms a multiprotein complex with CASP3 during genotoxic stress and inhibits its cleavage and activation [71, 72]. XIAP activates NFKB-dependent transcription via its NH2-terminal baculovirus inhibitor of apoptosis protein repeat (BIR) domain by activating SMAD signaling. SMAD signaling activates the expression of autophagy-related genes and the MAPK/JNK pathway. Conversely, the MAPK/JNK pathway can switch to the NFKB signaling pathway (Fig. 2) [73, 74]. Thus, TRIM8 prevents cell death upon genotoxic stress and radiotherapy by these novel mechanisms, suggesting that the high oncogenic potential of TRIM8 may support cancer cell viability [62].

Suppression of autophagy as a biological protective process against environmental and cellular stress has been investigated as a cancer therapy target, as it may predispose cancer cells to various treatments, such as exposure to DNA-damaging agents and radiation. The difficulty of acting directly on the components of autophagy—which play essential roles in normal cell physiology, and autophagy-related proteins participate in other cellular processes—could be reduced if there were a way to target cancer-promoting autophagy while allowing different types of autophagy to function efficiently. In principle, this can be achieved by targeting proteins with autophagy-specific activities rather than the core components of the machinery or lysosomal function to prevent complete autophagy. One proposed approach is to use the TRIM proteins due to their role in autophagy regulation, demonstrating the great potential of modulating TRIMs in oncogenesis or cancer progression.

### TRIM8 controls bipolar spindle formation and chromosomal stability

The formation of a bipolar spindle, which divides and separates the duplicated chromosomes during cell division, is one of the most critical processes in cell division. The controlled alignment of microtubules (MT) and the combined forces exerted by highly conserved motor proteins, including kinesins and dyneins, are also required to properly separate the duplicated chromosomes [75]. When duplicated chromosomes are not properly segregated due to defective formation of the bipolar spindle, chromosome mis-segregation and aneuploidy occur. Cancer cells typically exhibit a higher rate of chromosome mis-segregation and an aneuploid karyotype [75, 76]. E3 ubiquitin ligases are post-translational modifiers that facilitate the binding of ubiquitin to target proteins involved in the control of mitotic spindle machinery, including the TRIM protein family, whose dysregulation has been linked to a number of human diseases, including cancer [77]. A variety of TRIM proteins are important in mitotic and cell cycle transitions. In particular, interest in TRIM8 has increased dramatically in recent years. Studies have shown that TRIM8 is critical for the maintenance of genome integrity during cell division and the formation of the mitotic spindle machinery during mitosis [78]. TRIM8-silenced cells are responsible for a significant portion of the aneuploidy phenotype due to delayed progression through the G2/M phase of the cell cycle associated with centrosome and mitotic spindle abnormalities [78]. TRIM8 regulates cell cycle progression and mitosis by affecting cell cycle checkpoints and critical mitotic regulators and indirectly interacts with various motor microtubule-associated proteins (MAPs) such as kinesins [79].

Venuto et al. demonstrated that TRIM8 is found at the mitotic spindle during all phases of the cell cycle and interacts with KIFC1/HSET, KIF11/Kinesin-5/Eg5, KIF20B, and KIF2C, which are involved in the development of a bipolar spindle during mitosis, suggesting, that TRIM8 plays an essential role in determining cell polarity from the onset of centrosome duplication at the G1/S transition to the end of mitosis, where a cell divides into two identical daughter cells, a fundamental process in eukaryotic life mediated by microtubules and members of the kinesin family [79]. This physical contact between TRIM8 and KIF11 or KIFC1 is critical for proper microtubule assembly, ensuring the active structural configuration of these proteins and their mutual alignment along the mitotic spindle [80, 81]. KIFC1, an important member of the KIF14 superfamily in neurons with a specific minus-end directed motor, has also been associated with endocytic vesicle motility and cleavage [82], oocyte maturation [83], and long-distance transport of naked double-stranded DNA [84]. KIFC1 and KIF11 work together to promote microtubule aster formation, centrosome segregation, and proper spindle organization [85]. Impaired expression of KIF11 or KIFC1 is responsible for the abnormal spindle phenotype [86]. In particular, inhibition of KIF11 function by immunodepletion or knockdown of *KIF11* mRNA by small interfering RNA leads to cell cycle arrest in mitosis with monopolar spindle phenotype [86]. Considering the biological functions of KIF11 and KIFC1, TRIM8, with its E3 ubiquitin ligase activity, is likely involved in mitosis via ubiquitination of KIF11 and KIFC1 proteins in GSCs. KIF11 and KIFC1 are increased in GBM and are associated with the increased proliferation, self-renewal, and invasive behavior that are hallmarks of this brain tumor [80, 87]. KIF11 is increased in glioblastomas and is inversely related to overall survival. This protein promotes stem cell formation in glioma cells and increases cell proliferation and chemoresistance in malignant brain tumors [88, 89]. The Cancer Genome Atlas/TCGA data revealed that KIF11 is highly expressed in grade IV tissues compared with lower-grade and normal tissues. It is suggested that, in GBM, the E3 ubiquitin ligase function of TRIM8 is disrupted with KIF11 and KIFC1, resulting in increased expression of the latter proteins [90]. This finding implies that other TRIM proteins may have similar functions in transporting motor proteins or be transported as cargo within the cell.

Studies have shown that TRIM8 plays an important role in the progression of centrosome duplication. TRIM8 localizes to centrosomes and colocalizes with PLK1 (polo-like kinase 1), a human protein kinase with high sequence similarity to Cdc5 in *Drosophila*, and interacts directly with the centrosomal protein CEP170 [90]. PLK1, a key regulator in mitotic cell division, is involved in a number of critical processes, including mitotic entry, kinetochore–microtubule binding, and spindle formation [91]. This interaction primarily inhibits TRIM8 activity and delays mitotic progression, making cells more likely to arrest or be delayed in G2/M phase. Accumulation of cells arrested in the G2/M phase of the cell cycle leads to either initiation of the apoptotic pathway and activation of DNA damage responses or persistence of aneuploidy. The mechanisms of apoptosis mediated by silenced-TRIM8 cells require further investigation [92]. CEP170 localizes to both mother centrosomes during interphase and spindle microtubules during mitosis and plays a role in microtubule assembly and cell morphology determination [93].

Studies have shown that TRIM8 is also required for reliable chromosome segregation in mitosis. The process of chromosome segregation during mitosis is highly complex, and defects in this pathway can lead to mis-segregation and/or a non-integral set of 46 chromosomes. Knockdown of TRIM8 increases the rate of chromosomal instability and delays centrosome segregation, leading to an increase in aneuploid cells and micronucleus formation, demonstrating the essential role of TRIM8 in maintaining chromosomal integrity during mitosis [90]. Overall, deficiency of TRIM8-E3 ligase in glioma cells may promote carcinogenesis by promoting chromosome segregation defects during mitosis, leading to structural and non-euploid chromosome number aberrations, implying that TRIM8 may have tumor suppressor function during mitosis [94]. Chromosomal instability is a characteristic of human malignancies linked to poor prognosis, immune evasion, therapeutic resistance, and metastasis [95].

### TRIM8 as a target in chemoresistance

Drug resistance of tumors is a significant obstacle to cancer therapy [96, 97]. Understanding the signaling pathways is critical for determining the enzymes involved in chemoresistance in order to target them in combination therapies and make cells susceptible to standard chemotherapeutic agents. The ubiquitin–proteasome system has been recognized as a key player in a variety of physiological processes, including cell proliferation, autophagy, apoptosis, and DNA repair, all of which have been linked to carcinogenesis, cancer development, and drug resistance [98, 99]. Therefore, the use of proteasome inhibitors that alter the proteasome-mediated degradation pathway represents a new and promising method for treating human tumors with fewer side effects [99]. In particular, E3 ligases have attracted increasing attention in cancer and resistance research [100]. E3 ligase inhibitors are thought to specifically sensitize tumor cells to chemotherapeutic agents and radiotherapy by stabilizing or promoting the degradation of a subset of tumor suppressors or oncoproteins without affecting the activity of other proteins necessary for normal cell function [99, 100]. The most important type of E3 ligase is the really interesting new gene (RING) finger family, distinguished by its conserved RING domain. Other growing types of E3 ligases include the homologous to the E6AP carboxyl terminus/HECT type, the U-box type, and the RING-IBR-RING/RBR type, which are critical in drug resistance in several malignancies, including GBM. The TRIM protein family is a large subgroup of RING-type E3 ligases [101]. TRIM proteins act as both cancer driver and tumor suppressor proteins in regulating cell proliferation, depending on tumor type and deregulation processes. Many TRIM proteins are elevated in GBM (e.g., TRIMs 11, 14, 22, 25, 28, 32, 44, 59, and 65) [12, 102–108]. Abnormal overexpression of these TRIMs has been associated with poor prognosis and poor overall survival. In contrast, TRIMs 13, 16, 21, and 62 are potential tumor suppressors in a variety of malignancies, including GBM [11, 109]. TRIM8 is considered a cancer driver and tumor suppressor in controlling cell proliferation. The ability of TRIM8 to modulate the stability and activity of p53-mediated tumor suppressive activity is one of the reasons why it exerts a tumor suppressor function [48]. In addition, TRIM8 stimulates the degradation of MDM2, a primary cellular TP53 inhibitor, and directs the TP53 response toward growth arrest rather than apoptosis [48]. In general, patients with cancer with higher chemotherapy resistance have more mutations in the *TP53* gene or inactivation in its signaling pathway due to alterations in its regulators [110]. This is especially true for malignancies such as GBM, where the TP53 pathway is deregulated in 84% of patients, implying that reactivation of the TP53 pathway may be one of the most promising therapeutic approaches [111]. TRIM8 expression has been shown to correlate with increased TP53 activation and MDM2 instability in glioma tissues and cell lines, enhancing the effects of chemotherapeutic agents such as cisplatin and nutlin-3. In contrast, the silencing of TRIM8 correlates with the inactivation of TP53 and resistance to these chemotherapeutic agents. This suggests that TRIM8 levels play an essential role in TP53-mediated cellular responses to chemotherapeutic agents [15, 51]. Another example of TRIM8 activity in chemosensitivity is in anaplastic thyroid carcinoma/ATC, where downregulation of TRIM8 essentially correlates with overexpression of *MIR182* in human ATC tissues. Qin et al. found that *MIR182* promoted tumor development by suppressing TRIM8 expression and contributed to the chemoresistance of human ATC to standard chemotherapeutic agents such as cisplatin. On the basis of these results, it was concluded that *MIR182*-TRIM8 could be a therapeutic target for the treatment of chemoresistant human papillary thyroid carcinoma [112]. Tullo et al. found that TRIM8 is a target of *MIR17-5p* and *MIR106B*-*5p*, both of which are overexpressed in chemo-/radioresistant cancers such as ccRCC and GBM. MYCN promotes carcinogenesis by activating *MIR17*-*5p* and *MIR106B*-*5p*, and this oncogene is inhibited by *MIR34A*, whose expression is induced by TP53. Of note, silencing of *MIR17*-*5p* and *MIR106B*-*5p* enhances TRIM8 expression. It leads to the restoration of tumor suppressor activity of TP53 in a TRIM8-dependent manner, thereby restoring the sensitivity of cells to clinically used chemotherapeutic agents such as sorafenib and axitinib, which are used as second-line treatments for advanced renal cell carcinoma [51, 113, 114].

Chemosensitization by TRIM8 was also observed in SW620 and SW480 cells. SW620 and SW480 cells are two different colon cancer cell lines with different levels of TP53 protein [115, 116]. SW620 cells have wild-type TP53 protein, whereas SW480 cells lack TP53 protein. SW620 cells with higher TRIM8 expression were more susceptible to the chemotherapeutic agent 5-fluorouracil, whereas silencing TRIM8 increased SW620 cell survival. However, this was not the case for SW480 cells. Therefore, TRIM8 was shown to increase the susceptibility of CRC cells to the above chemotherapeutic agent in a TP53-dependent manner [115]. It is probably important to evaluate the tumor suppressive and oncogenic activity of TRIM8 in relation to the molecular status of p53 because, if p53 is mutated, TRIM8 expression is partially oncogenic [48].

The effects of TRIM8 on the stability and activity of the oncogenic form of the TP63 transcription factor, ∆Np63, which shares structural similarities with the tumor suppressor TP53, reveal another vital role for TRIM8 in sensitizing cells to chemotherapeutic agents [117]. Numerous studies have shown that TP63 plays an important role in cancer development, resistance to chemotherapy, metastasis, and survival of cancer cells. TP63 is overexpressed in various types of malignant tumors, suggesting that it confers a selective growth advantage to cancer cells. Overexpression of TP63 in oral squamous cell carcinoma/OSCC has recently been shown to be a potential marker of radioresistance and a predictor of poor prognosis [117–119]. Tullo et al. found that TRIM8 enhances the tumor suppressor activity of TP53 and decreases the expression of the TP63 protein in a manner that is dependent on both the ubiquitin–proteasome system and caspase 1 (CASP1) [118]. In addition, studies have shown that TP63 decreases *TRIM8* gene expression by inhibiting the TP53-directed transcriptional program of TRIM8, indicating the presence of a negative feedback loop. These results suggest that increasing TRIM8 activity could provide therapeutic benefits and improve the treatment of chemoresistant malignancies, particularly GBM [117, 120].

### TRIM8 as an inflammation inducer

The relationship between inflammation and cancer has attracted much attention in recent decades. The importance of inflammation in gliomas is less evident than in other cancers, especially at the onset. The transcription factor NFKB induces the expression of genes involved in many aspects of the innate and adaptive immune system and is one of the most important molecules in triggering chronic inflammation as a hallmark and cause of cancer [121, 122]. Constitutive NFKB activation is a common phenomenon in GBM, as in many other malignancies. Inflammation has been reported to promote mesenchymal differentiation, maintenance of cancer stem-like cells, and radiation resistance [123], and it also plays a key role in several other active carcinogenic processes in GBM [124].

Mutations or overexpression of NFKB signaling components such as TNF receptor-associated factor 2 (TRAF2) and TNFRSF1A associated via death domain (TRADD) are rare in tumors, suggesting that abnormal activation of NFKB signaling in GBM may be due to pathway dysregulation or oncogenes [125]. TRIM proteins are involved in the development of various malignancies by affecting a number of biological processes, including modulation of NFKB transcriptional activity. TRIM40 is downregulated in the gastrointestinal tract during carcinogenesis, which inhibits NFKB activity via neddylation of IKBKG, a critical regulator of NFKB activation, and consequently causes inhibition of NFKB activity [126].

In recent years, there has been a surge of interest in TRIM8 as an activator of NFKB signaling. At least two subcellular sites (the cytoplasm and nucleus) have been identified where the ubiquitin ligase activity of TRIM8 is required to activate the NFKB pathway. Nucleocytoplasmic transport of TRIM8 is necessary for positive control of NFKB activation [127]. TRIM8 reduces the nuclear localization of endogenous PIAS3 and its RING domain is required for this function. This translocation from the nucleus to the cytoplasm impairs the negative regulation of NFKB at the RELA/p65 subunit through the activity of PIAS3, and enhances NFKB transcription factor dimerization and activation of NFKB-responsive genes [28, 128].

TRIM8 also functions as a positive regulator of cytokine-induced NFKB activation in the cytoplasm. Wang et al. demonstrated that TRIM8 promotes K63-linked polyubiquitination of MAP3K7/TAK1 at Lys158, but not K48-linked polyubiquitination after activation of surface receptors such as TNF or interleukin 1 receptor (IL1R) [29]. Activated MAP3K7/TAK1 is required for the IKK complex-induced NFKB pathway activation. The IKK complex phosphorylates NFKBIA/IKBA protein, leading to ubiquitination and degradation by the 26S proteasome and translocation of NFKB to the nucleus, allowing the activation of NFKB-responsive genes [29, 129]. The long noncoding RNA *GNAS-AS1/Nespas* inhibits TRIM8-induced Lys63-linked polyubiquitination of MAP3K7/TAK1, suppressing inflammatory cytokine production and NFKB signaling activation [130]. Deregulated NFKB activation is a common phenomenon in glioblastoma; its activity is a significant driver of the malignant phenotype, ranging from tumor growth and invasion to the maintenance of cancer stem-like cells, suppression of programmed cell death, and resistance to radiotherapy [131].

A well-known function of NFKB is the regulation of inflammatory responses by controlling the expression of proinflammatory genes and activities in innate and adaptive immune cells. Not surprisingly, NFKB expression is a marker of inflammation and has attracted considerable attention in the field of inflammation-related cancers [132, 133]. Because NFKB has been identified as a driver of several features of gliomagenesis and treatment tolerance, the NFKB signaling network is now an attractive therapeutic target. Thanks to recent advances in drug discovery, a variety of drugs targeting NFKB are now available, and several of them have shown promise in preclinical studies, either alone or in combination with temozolomide, a first-line chemotherapeutic agent in GBM [134, 135]. Research has shown that inhibition of NFKB in combination with temozolomide can synergistically enhance glioma cell suppression. However, further research is needed to elucidate the activity of TRIM8 and establish links between inflammation and carcinogenesis.

## Conclusion

GBM is the most common type of brain tumor in adults worldwide. Among the newly identified glioma-associated genes, interest in TRIM8 has increased dramatically in recent years. TRIM8 is an E3 ubiquitin ligase involved in many biological processes such as autophagy, apoptosis, and differentiation, all of which are required to maintain cellular homeostasis and thus regulate most signal transduction pathways. Our study suggests that TRIM8 plays a role in GBM carcinogenesis by positively regulating key cellular signaling pathways such as NFKB and JAK-STAT, which effectively enhance the stem-like property of GSCs and potentially provide clues for innovative treatments. TRIM8 also exerts its anticancer effect by potentiating tumor suppressor TP53 through interaction with MDM2, an important inhibitor of TP53, and, conversely, by suppressing the activity of the oncogenic protein ΔNp63. This suggests that TRIM8 confers a selective growth disadvantage to cancer cells, and enhancing TRIM8 activity could provide therapeutic benefits and improve the treatment of chemoresistant tumors. In this study, we summarized the dual role of TRIM8 in cancer as an oncogene or tumor suppressor gene in regulating autophagy, controlling bipolar spindle formation and chromosomal stability, regulating chemoresistance, and triggering inflammation. We believe that it is critical to understand how TRIM8-associated axes can be further modulated for the development of cancer therapeutics, as this could provide new insights into understanding the pathophysiology of GBM cancer and the development of therapeutic targets. The use of E3 ligase inhibitors or targeted protein degraders [Molecular Glues, Proteolysis Targeting Chimeras (PROTACs), and Hydrophobic Tag (HyT)] of TRIM8 would be a suitable way to regulate the amount of TRIM8 protein and thus an exciting possibility for therapeutic intervention. Enhancement of the p53-mediated tumor suppressor activity of TRIM8 in the tumors with wild-type p53 could be another potential therapeutic.

## Data Availability

Not applicable.

## Abbreviations

CSC: Cancer stem cell
GBM: Glioblastoma
TRIM: Tripartite motif protein family
RBCC: RING domain, B-box motif, and coiled-coil domain
TRIM8/GERP: Tripartite motif containing 8
GSCs: Glioblastoma stem cells
PIAS: Protein inhibitor of activated STAT
STAT: Signal transducer and activator of transcription
TNF/TNFα: Tumor necrosis factor
IL1B/IL -1β: Interleukin 1 beta
MAP3K7/TAK1: Mitogen-activated protein kinase kinase kinase 7
SOCS1: Suppressor of cytokine signaling 1
KIRs: Kinase inhibitory domains
IL6: Interleukin 6
EGFR: Epidermal growth factor receptor
VEGF: Vascular endothelial growth factor
ccRCC: Clear cell renal cell carcinoma
TFs: Transcription factors
IKBKG/NEMO/IKKγ: Inhibitor of nuclear factor kappa B kinase regulatory subunit gamma
IKK: IκB kinase complex
XIAP/IAP3: X-linked inhibitor of apoptosis
BIR: Baculovirus inhibitor of apoptosis protein repeat
MT: Microtubules
MAPs: Microtubule-associated proteins
Plk1: Polo-like kinase 1
RING: Really interesting new gene
TRAF2: TNF receptor-associated factor 2
TRADD: TNFRSF1A associated via death domain

## References

[1] Mafi A, Rahmati A, BabaeiAghdam Z, Salami R, Salami M, Vakili O (2022). Recent insights into the microRNA-dependent modulation of gliomas from pathogenesis to diagnosis and treatment. Cell Mol Biol Lett.

[2] Fang Y, Zhang Z (2020). Arsenic trioxide as a novel anti-glioma drug: a review. Cell Mol Biol Lett.

[3] Datsi A, Sorg RV (2021). Dendritic cell vaccination of glioblastoma: road to success or dead end. Front Immunol.

[4] Noch EK, Ramakrishna R, Magge R (2018). Challenges in the treatment of glioblastoma: multisystem mechanisms of therapeutic resistance. World Neurosurg.

[5] Yin Y, Zhong J, Li S-W, Li J-Z, Zhou M, Chen Y (2016). TRIM11, a direct target of miR-24-3p, promotes cell proliferation and inhibits apoptosis in colon cancer. Oncotarget.

[6] Chen Y, Li L, Qian X, Ge Y, Xu G (2017). High expression of TRIM11 correlates with poor prognosis in patients with hepatocellular carcinoma. Clin Res Hepatol Gastroenterol.

[7] Hatakeyama S (2011). TRIM proteins and cancer. Nat Rev Cancer.

[8] Wang B, Wang G, Wang Q, Zhu Z, Wang Y, Chen K (2019). Silencing of TRIM11 suppresses the tumorigenicity of chordoma cells through improving the activity of PHLPP1/AKT. Cancer Cell Int.

[9] Bhaduri U, Merla G (2020). Rise of TRIM8: a molecule of duality. Mol Ther Nucleic Acids.

[10] Reymond A, Meroni G, Fantozzi A, Merla G, Cairo S, Luzi L (2001). The tripartite motif family identifies cell compartments. Embo J.

[11] Vincent SR, Kwasnicka DA, Fretier P (2000). A novel RING finger-B box-coiled-coil protein, GERP. Biochem Biophys Res Commun.

[12] Di K, Linskey ME, Bota DA (2013). TRIM11 is overexpressed in high-grade gliomas and promotes proliferation, invasion, migration and glial tumor growth. Oncogene.

[13] Marzano F, Guerrini L, Pesole G, Sbisà E, Tullo A (2021). Emerging roles of TRIM8 in health and disease. Cells.

[14] Zhang C, Mukherjee S, Tucker-Burden C, Ross JL, Chau MJ, Kong J (2017). TRIM8 regulates stemness in glioblastoma through PIAS3-STAT3. Mol Oncol.

[15] Micale L, Fusco C, Fontana A, Barbano R, Augello B, De Nittis P (2015). TRIM8 downregulation in glioma affects cell proliferation and it is associated with patients survival. BMC Cancer.

[16] Lathia JD, Mack SC, Mulkearns-Hubert EE, Valentim CLL, Rich JN (2015). Cancer stem cells in glioblastoma. Genes Dev.

[17] Alkhaibary A, Alassiri AH, AlSufiani F, Alharbi MA (2019). Ki-67 labeling index in glioblastoma; does it really matter?. Hematol Oncol Stem Cell Ther.

[18] Okumura F, Matsunaga Y, Katayama Y, Nakayama KI, Hatakeyama S (2010). TRIM8 modulates STAT3 activity through negative regulation of PIAS3. J Cell Sci.

[19] Hu X, Li J, Fu M, Zhao X, Wang W (2021). The JAK/STAT signaling pathway: from bench to clinic. Signal Transduct Target Ther..

[20] Yagil Z, Nechushtan H, Kay G, Yang CM, Kemeny DM, Razin E (2010). The enigma of the role of protein inhibitor of activated STAT3 (PIAS3) in the immune response. Trends Immunol.

[21] Levy C, Nechushtan H, Razin E (2002). A new role for the STAT3 inhibitor, PIAS3: a repressor of microphthalmia transcription factor *. J Biol Chem.

[22] Galoczova M, Coates P, Vojtesek B (2018). STAT3, stem cells, cancer stem cells and p63. Cell Mol Biol Lett.

[23] Guryanova OA, Wu Q, Cheng L, Lathia JD, Huang Z, Yang J (2011). Nonreceptor tyrosine kinase BMX maintains self-renewal and tumorigenic potential of glioblastoma stem cells by activating STAT3. Cancer Cell.

[24] Kim E, Kim M, Woo D-H, Shin Y, Shin J, Chang N (2013). Phosphorylation of EZH2 activates STAT3 signaling via STAT3 methylation and promotes tumorigenicity of glioblastoma stem-like cells. Cancer Cell.

[25] Herrmann A, Cherryholmes G, Schroeder A, Phallen J, Alizadeh D, Xin H (2014). TLR9 is critical for glioma stem cell maintenance and targeting. Can Res.

[26] Vázquez-Arreguín K, Tantin D (2016). The Oct1 transcription factor and epithelial malignancies: old protein learns new tricks. Biochem Biophys Acta.

[27] Jaworska AM, Wlodarczyk NA, Mackiewicz A, Czerwinska P (2020). The role of TRIM family proteins in the regulation of cancer stem cell self-renewal. Stem Cells.

[28] Tomar D, Sripada L, Prajapati P, Singh R, Singh AK, Singh R (2012). Nucleo-cytoplasmic trafficking of TRIM8, a novel oncogene, is involved in positive regulation of TNF induced NF-κB pathway. PLoS ONE.

[29] Li Q, Yan J, Mao AP, Li C, Ran Y, Shu HB (2011). Tripartite motif 8 (TRIM8) modulates TNFα- and IL-1β-triggered NF-κB activation by targeting TAK1 for K63-linked polyubiquitination. Proc Natl Acad Sci U S A.

[30] Li Q, Yan J, Mao AP, Li C, Ran Y, Shu HB (2011). Tripartite motif 8 (TRIM8) modulates TNFα- and IL-1β-triggered NF-κB activation by targeting TAK1 for K63-linked polyubiquitination. Proc Natl Acad Sci USA.

[31] Jang HD, Yoon K, Shin YJ, Kim J, Lee SY (2004). PIAS3 suppresses NF-κB-mediated transcription by interacting with the p65/RelA subunit *. J Biol Chem.

[32] Liu X, Lei X, Wang J, Hong T (2011). Identification A novel protein TRIM38 that activate NF-kappaB signaling pathways. (Zhonghua Shiyan he Linchuang Bingduxue Zazhi) Chin J Exp Clin Virol.

[33] Wu Y-D, Zhou B (2010). TNF-α/NF-κB/Snail pathway in cancer cell migration and invasion. Br J Cancer.

[34] Toniato E, Chen XP, Losman J, Flati V, Donahue L, Rothman P (2002). TRIM8/GERP RING finger protein interacts with SOCS-1 *. J Biol Chem.

[35] Aringer M, Cheng A, Nelson JW, Chen M, Sudarshan C, Zhou YJ (1999). Janus kinases and their role in growth and disease. Life Sci.

[36] Tamir I, Dal Porto JM, Cambier JC (2000). Cytoplasmic protein tyrosine phosphatases SHP-1 and SHP-2: regulators of B cell signal transduction. Curr Opin Immunol.

[37] Croker BA, Kiu H, Nicholson SE (2008). SOCS regulation of the JAK/STAT signalling pathway. Semin Cell Dev Biol.

[38] Liau NPD, Laktyushin A, Lucet IS, Murphy JM, Yao S, Whitlock E (2018). The molecular basis of JAK/STAT inhibition by SOCS1. Nat Commun.

[39] Wang H, Lathia JD, Wu Q, Wang J, Li Z, Heddleston JM (2009). Targeting interleukin 6 signaling suppresses glioma stem cell survival and tumor growth. Stem Cells.

[40] Fan X, Khaki L, Zhu TS, Soules ME, Talsma CE, Gul N (2010). NOTCH pathway blockade depletes CD133-positive glioblastoma cells and inhibits growth of tumor neurospheres and xenografts. Stem Cells.

[41] Banerjee K, Resat H (2016). Constitutive activation of STAT3 in breast cancer cells: a review. Int J Cancer.

[42] Network CGAR (2008). Comprehensive genomic characterization defines human glioblastoma genes and core pathways. Nature.

[43] Lu S, Wang S, Geng S, Ma S, Liang Z, Jiao B (2012). Increased expression of microRNA-17 predicts poor prognosis in human glioma. J Biomed Biotechnol.

[44] Bomben R, Gobessi S, Dal Bo M, Volinia S, Marconi D, Tissino E (2012). The miR-17∼92 family regulates the response to Toll-like receptor 9 triggering of CLL cells with unmutated IGHV genes. Leukemia.

[45] Okumura F, Okumura AJ, Matsumoto M, Nakayama KI, Hatakeyama S (2011). TRIM8 regulates Nanog via Hsp90β-mediated nuclear translocation of STAT3 in embryonic stem cells. Biochim Biophys Acta.

[46] Setati MM, Prinsloo E, Longshaw VM, Murray PA, Edgar DH, Blatch GL (2010). Leukemia inhibitory factor promotes Hsp90 association with STAT3 in mouse embryonic stem cells. IUBMB Life.

[47] Forte IM, Indovina P, Iannuzzi CA, Cirillo D, Di Marzo D, Barone D (2019). Targeted therapy based on p53 reactivation reduces both glioblastoma cell growth and resistance to temozolomide. Int J Oncol.

[48] Caratozzolo MF, Micale L, Turturo MG, Cornacchia S, Fusco C, Marzano F (2012). TRIM8 modulates p53 activity to dictate cell cycle arrest. Cell Cycle.

[49] Elabd S, Meroni G, Blattner C (2016). TRIMming p53’s anticancer activity. Oncogene.

[50] Caratozzolo MF, Valletti A, Gigante M, Aiello I, Mastropasqua F, Marzano F (2014). TRIM8 anti-proliferative action against chemo-resistant renal cell carcinoma. Oncotarget.

[51] Mastropasqua F, Marzano F, Valletti A, Aiello I, Di Tullio G, Morgano A (2017). TRIM8 restores p53 tumour suppressor function by blunting N-MYC activity in chemo-resistant tumours. Mol Cancer.

[52] Patel JH, Loboda AP, Showe MK, Showe LC, McMahon SB (2004). Analysis of genomic targets reveals complex functions of MYC. Nat Rev Cancer.

[53] Choi YJ, Lin C-P, Ho JJ, He X, Okada N, Bu P (2011). miR-34 miRNAs provide a barrier for somatic cell reprogramming. Nat Cell Biol.

[54] Bai X, Zhang Y-L, Liu L-N (2020). Inhibition of TRIM8 restrains ischaemia-reperfusion-mediated cerebral injury by regulation of NF-κB activation associated inflammation and apoptosis. Exp Cell Res.

[55] Seong BKA, Dharia NV, Lin S, Donovan KA, Chong S, Robichaud A (2021). TRIM8 modulates the EWS/FLI oncoprotein to promote survival in Ewing sarcoma. Cancer Cell.

[56] Aynaud M-M, Mirabeau O, Gruel N, Grossetête S, Boeva V, Durand S (2020). Transcriptional programs define intratumoral heterogeneity of Ewing sarcoma at single-cell resolution. Cell Rep.

[57] Delattre O, Zucman J, Plougastel B, Desmaze C, Melot T, Peter M (1992). Gene fusion with an ETS DNA-binding domain caused by chromosome translocation in human tumours. Nature.

[58] Mulcahy Levy JM, Thorburn A (2020). Autophagy in cancer: moving from understanding mechanism to improving therapy responses in patients. Cell Death Differ.

[59] Gschwind A, Marx C, Just MD, Severin P, Behring H, Marx-Blümel L (2022). Tight association of autophagy and cell cycle in leukemia cells. Cell Mol Biol Lett.

[60] Ylä-Anttila P (2021). Autophagy receptors as viral targets. Cell Mol Biol Lett.

[61] Jin L, Yuan F, Dai G, Yao Q, Xiang H, Wang L (2020). Blockage of O-linked GlcNAcylation induces AMPK-dependent autophagy in bladder cancer cells. Cell Mol Biol Lett.

[62] Roy M, Tomar D, Singh K, Lakshmi S, Prajapati P, Bhatelia K (2018). TRIM8 regulated autophagy modulates the level of cleaved Caspase-3 subunit to inhibit genotoxic stress induced cell death. Cell Signal.

[63] Bell JL, Malyukova A, Holien JK, Koach J, Parker MW, Kavallaris M (2012). TRIM16 acts as an E3 ubiquitin ligase and can heterodimerize with other TRIM family members. PLoS ONE.

[64] Kawadler H, Yang X (2006). Lys63-linked polyubiquitin chains: linking more than just ubiquitin. Cancer Biol Ther.

[65] Tenno T, Fujiwara K, Tochio H, Iwai K, Morita EH, Hayashi H (2004). Structural basis for distinct roles of Lys63- and Lys48-linked polyubiquitin chains. Genes Cells.

[66] Nivon M, Richet E, Codogno P, Arrigo A-P, Kretz-Remy C (2009). Autophagy activation by NFκB is essential for cell survival after heat shock. Autophagy.

[67] Copetti T, Bertoli C, Dalla E, Demarchi F, Schneider C (2009). p65/RelA modulates BECN1 transcription and autophagy. Mol Cell Biol.

[68] Mandell MA, Saha B, Thompson TA (2020). The tripartite Nexus: autophagy, cancer, and tripartite motif-containing protein family members. Front Pharmacol.

[69] Zhong Z, Umemura A, Sanchez-Lopez E, Liang S, Shalapour S, Wong J (2016). NF-κB restricts inflammasome activation via elimination of damaged mitochondria. Cell.

[70] Jamil S, Lam I, Majd M, Tsai S-H, Duronio V (2015). Etoposide induces cell death via mitochondrial-dependent actions of p53. Cancer Cell Int.

[71] Riedl SJ, Renatus M, Schwarzenbacher R, Zhou Q, Sun C, Fesik SW (2001). Structural basis for the inhibition of caspase-3 by XIAP. Cell.

[72] Bratton SB, Lewis J, Butterworth M, Duckett CS, Cohen GM (2002). XIAP inhibition of caspase-3 preserves its association with the Apaf-1 apoptosome and prevents CD95- and Bax-induced apoptosis. Cell Death Differ.

[73] Hofer-Warbinek R, Schmid JA, Stehlik C, Binder BR, Lipp J, de Martin R (2000). Activation of NF-kappa B by XIAP, the X chromosome-linked inhibitor of apoptosis, in endothelial cells involves TAK1. J Biol Chem.

[74] Dubrez-Daloz L, Dupoux A, Cartier J (2008). IAPs: more than just inhibitors of apoptosis proteins. Cell Cycle.

[75] Hinchcliffe EH (2011). The centrosome and bipolar spindle assembly: does one have anything to do with the other?. Cell Cycle.

[76] Sivakumar S, Gorbsky GJ (2015). Spatiotemporal regulation of the anaphase-promoting complex in mitosis. Nat Rev Mol Cell Biol.

[77] Venuto S, Merla G (2019). E3 ubiquitin ligase TRIM proteins, cell cycle and mitosis. Cells.

[78] Esposito JE, De Iuliis V, Avolio F, Liberatoscioli E, Pulcini R, Di Francesco S (2022). Dissecting the functional role of the TRIM8 protein on cancer pathogenesis. Cancers.

[79] Venuto S, Monteonofrio L, Cozzolino F, Monti M, Appolloni I, Mazza T (2020). TRIM8 interacts with KIF11 and KIFC1 and controls bipolar spindle formation and chromosomal stability. Cancer Lett.

[80] Venere M, Horbinski C, Crish JF, Jin X, Vasanji A, Major J (2015). The mitotic kinesin KIF11 is a driver of invasion, proliferation, and self-renewal in glioblastoma. Sci Transl Med..

[81] Labonté D, Thies E, Pechmann Y, Groffen AJ, Verhage M, Smit AB (2013). TRIM3 regulates the motility of the kinesin motor protein KIF21B. PLoS ONE.

[82] Nath S, Bananis E, Sarkar S, Stockert RJ, Sperry AO, Murray JW (2007). Kif5B and Kifc1 interact and are required for motility and fission of early endocytic vesicles in mouse liver. Mol Biol Cell.

[83] Hall VJ, Compton D, Stojkovic P, Nesbitt M, Herbert M, Murdoch A (2007). Developmental competence of human in vitro aged oocytes as host cells for nuclear transfer. Hum Reprod.

[84] Farina F, Pierobon P, Delevoye C, Monnet J, Dingli F, Loew D (2013). Kinesin KIFC1 actively transports bare double-stranded DNA. Nucleic Acids Res.

[85] Bhaduri U, Merla G (2020). Rise of TRIM8: a molecule of duality. Mol Ther Nucleic Acids.

[86] Sarli V, Giannis A (2008). Targeting the kinesin spindle protein: basic principles and clinical implications. Clin Cancer Res.

[87] Wu J, Wang X, Yuan X, Shan Q, Wang Z, Wu Y (2021). Kinesin family member C1 increases temozolomide resistance of glioblastoma through promoting DNA damage repair. Cell Transplant.

[88] Liu B, Zhang G, Cui S, Du G (2022). Upregulation of KIF11 in TP53 mutant glioma promotes tumor stemness and drug resistance. Cell Mol Neurobiol.

[89] Kim N, Song K (2013). KIFC1 is essential for bipolar spindle formation and genomic stability in the primary human fibroblast IMR-90 cell. Cell Struct Funct.

[90] Venuto S, Monteonofrio L, Cozzolino F, Monti M, Appolloni I, Mazza T (2020). TRIM8 interacts with KIF11 and KIFC1 and controls bipolar spindle formation and chromosomal stability. Cancer Lett.

[91] Zhang Z, Chen C, Ma L, Yu Q, Li S, Abbasi B (2017). Plk1 is essential for proper chromosome segregation during meiosis I/meiosis II transition in pig oocytes. Reprod Biol Endocrinol.

[92] Tarasov KV, Tarasova YS, Tam WL, Riordon DR, Elliott ST, Kania G (2008). B-MYB is essential for normal cell cycle progression and chromosomal stability of embryonic stem cells. PLoS ONE.

[93] Bärenz F, Kschonsak YT, Meyer A, Jafarpour A, Lorenz H, Hoffmann I (2018). Ccdc61 controls centrosomal localization of Cep170 and is required for spindle assembly and symmetry. Mol Biol Cell.

[94] Cambiaghi V, Giuliani V, Lombardi S, Marinelli C, Toffalorio F, Pelicci PG (2012). TRIM proteins in cancer. Adv Exp Med Biol.

[95] Bakhoum SF, Cantley LC (2018). The multifaceted role of chromosomal instability in cancer and its microenvironment. Cell.

[96] Khanna A, Mahalingam K, Chakrabarti D, Periyasamy G (2011). Ets-1 expression and gemcitabine chemoresistance in pancreatic cancer cells. Cell Mol Biol Lett.

[97] Maleki Dana P, Sadoughi F, Asemi Z, Yousefi B (2022). The role of polyphenols in overcoming cancer drug resistance: a comprehensive review. Cell Mol Biol Lett.

[98] Marzano F, Caratozzolo MF, Pesole G, Sbisà E, Tullo A (2021). TRIM proteins in colorectal cancer: TRIM8 as a promising therapeutic target in chemo resistance. Biomedicines.

[99] Yang L, Chen J, Huang X, Zhang E, He J, Cai Z (2018). Novel insights into E3 ubiquitin ligase in cancer chemoresistance. Am J Med Sci.

[100] Liu J, Shaik S, Dai X, Wu Q, Zhou X, Wang Z (2015). Targeting the ubiquitin pathway for cancer treatment. Biochem Biophys Acta.

[101] Yang Q, Zhao J, Chen D, Wang Y (2021). E3 ubiquitin ligases: styles, structures and functions. Mol Biomed.

[102] Feng S, Cai X, Li Y, Jian X, Zhang L, Li B (2019). Tripartite motif-containing 14 (TRIM14) promotes epithelial-mesenchymal transition via ZEB2 in glioblastoma cells. J Exp Clin Cancer Res.

[103] Ji J, Ding K, Luo T, Zhang X, Chen A, Zhang D (2021). TRIM22 activates NF-κB signaling in glioblastoma by accelerating the degradation of IκBα. Cell Death Differ.

[104] Liu Y, Tao S, Liao L, Li Y, Li H, Li Z (2020). TRIM25 promotes the cell survival and growth of hepatocellular carcinoma through targeting Keap1-Nrf2 pathway. Nat Commun.

[105] Su C, Li H, Gao W (2018). TRIM28 is overexpressed in glioma and associated with tumor progression. Onco Targets Ther.

[106] Cai Y, Gu WT, Cheng K, Jia PF, Li F, Wang M (2021). Knockdown of TRIM32 inhibits tumor growth and increases the therapeutic sensitivity to temozolomide in glioma in a p53-dependent and -independent manner. Biochem Biophys Res Commun.

[107] Zhou X, Yang Y, Ma P, Wang N, Yang D, Tu Q (2019). TRIM44 is indispensable for glioma cell proliferation and cell cycle progression through AKT/p21/p27 signaling pathway. J Neurooncol.

[108] Sang Y, Li Y, Song L, Alvarez AA, Zhang W, Lv D (2018). TRIM59 promotes gliomagenesis by inhibiting TC45 dephosphorylation of STAT3. Cancer Res.

[109] Marshall GM, Bell JL, Koach J, Tan O, Kim P, Malyukova A (2010). TRIM16 acts as a tumour suppressor by inhibitory effects on cytoplasmic vimentin and nuclear E2F1 in neuroblastoma cells. Oncogene.

[110] Huang Y, Liu N, Liu J, Liu Y, Zhang C, Long S (2019). Mutant p53 drives cancer chemotherapy resistance due to loss of function on activating transcription of PUMA. Cell Cycle.

[111] Zhang Y, Dube C, Gibert M, Cruickshanks N, Wang B, Coughlan M (2018). The p53 pathway in glioblastoma. Cancers.

[112] Liu Y, Zhang B, Shi T, Qin H (2017). miR-182 promotes tumor growth and increases chemoresistance of human anaplastic thyroid cancer by targeting tripartite motif 8. Onco Targets Ther.

[113] Motzer RJ, Escudier B, Tomczak P, Hutson TE, Michaelson MD, Negrier S (2013). Axitinib versus sorafenib as second-line treatment for advanced renal cell carcinoma: overall survival analysis and updated results from a randomised phase 3 trial. Lancet Oncol.

[114] Bielecka ZF, Czarnecka AM, Solarek W, Kornakiewicz A, Szczylik C (2014). Mechanisms of acquired resistance to tyrosine kinase inhibitors in clear-cell renal cell carcinoma (ccRCC). Curr Signal Transduct Ther.

[115] Ni M, Wang Y, Xie L (2016). TRIM8 regulates the chemoresistance of colorectal cancer in a p53-dependent manner. Oncol Lett.

[116] Lamy V, Bousserouel S, Gossé F, Minker C, Lobstein A, Raul F (2010). p53 Activates either survival or apoptotic signaling responses in lupulone-treated human colon adenocarcinoma cells and derived metastatic cells. Transl Oncol.

[117] Moergel M, Abt E, Stockinger M, Kunkel M (2010). Overexpression of p63 is associated with radiation resistance and prognosis in oral squamous cell carcinoma. Oral Oncol.

[118] Caratozzolo MF, Marzano F, Abbrescia DI, Mastropasqua F, Petruzzella V, Calabrò V (2019). TRIM8 blunts the pro-proliferative action of ΔNp63α in a p53 wild-type background. Front Oncol.

[119] Matin RN, Chikh A, Chong SL, Mesher D, Graf M, Sanza P (2013). p63 is an alternative p53 repressor in melanoma that confers chemoresistance and a poor prognosis. J Exp Med.

[120] Loljung L, Coates PJ, Nekulova M, Laurell G, Wahlgren M, Wilms T (2014). High expression of p63 is correlated to poor prognosis in squamous cell carcinoma of the tongue. J Oral Pathol Med.

[121] Hosseinalizadeh H, Mahmoodpour M, Samadani AA, Roudkenar MH (2022). The immunosuppressive role of indoleamine 2, 3-dioxygenase in glioblastoma: mechanism of action and immunotherapeutic strategies. Med Oncol.

[122] Taniguchi K, Karin M (2018). NF-κB, inflammation, immunity and cancer: coming of age. Nat Rev Immunol.

[123] Bhat KP, Balasubramaniyan V, Vaillant B, Ezhilarasan R, Hummelink K, Hollingsworth F (2013). Mesenchymal differentiation mediated by NF-κB promotes radiation resistance in glioblastoma. Cancer Cell.

[124] Nagai S, Washiyama K, Kurimoto M, Takaku A, Endo S, Kumanishi T (2002). Aberrant nuclear factor-κB activity and its participation in the growth of human malignant astrocytoma. J Neurosurg.

[125] Verhaak RG, Hoadley KA, Purdom E, Wang V, Qi Y, Wilkerson MD (2010). Integrated genomic analysis identifies clinically relevant subtypes of glioblastoma characterized by abnormalities in PDGFRA, IDH1, EGFR, and NF1. Cancer Cell.

[126] Noguchi K, Okumura F, Takahashi N, Kataoka A, Kamiyama T, Todo S (2011). TRIM40 promotes neddylation of IKKγ and is downregulated in gastrointestinal cancers. Carcinogenesis.

[127] Marzano F, Guerrini L, Pesole G, Sbisà E, Tullo A (2021). Emerging roles of TRIM8 in health and disease. Cells.

[128] Okumura F, Matsunaga Y, Katayama Y, Nakayama KI, Hatakeyama S (2010). TRIM8 modulates STAT3 activity through negative regulation of PIAS3. J Cell Sci.

[129] Takaesu G, Surabhi RM, Park KJ, Ninomiya-Tsuji J, Matsumoto K, Gaynor RB (2003). TAK1 is critical for IkappaB kinase-mediated activation of the NF-kappaB pathway. J Mol Biol.

[130] Deng Y, Chen D, Wang L, Gao F, Jin B, Lv H (2019). Silencing of long noncoding RNA Nespas aggravates microglial cell death and neuroinflammation in ischemic stroke. Stroke.

[131] Soubannier V, Stifani S (2017). NF-κB signalling in glioblastoma. Biomedicines.

[132] Liu T, Zhang L, Joo D, Sun SC (2017). NF-κB signaling in inflammation. Signal Transduct Target Ther.

[133] Colotta F, Allavena P, Sica A, Garlanda C, Mantovani A (2009). Cancer-related inflammation, the seventh hallmark of cancer: links to genetic instability. Carcinogenesis.

[134] Singh N, Miner A, Hennis L, Mittal S (2021). Mechanisms of temozolomide resistance in glioblastoma—a comprehensive review. Cancer Drug Resist.

[135] Stupp R, Mason WP, van den Bent MJ, Weller M, Fisher B, Taphoorn MJ (2005). Radiotherapy plus concomitant and adjuvant temozolomide for glioblastoma. N Engl J Med.

